# 

**Published:** 1873-05

**Authors:** 


BOOKS and pamphlets will be noticed in our next number.

*List of Payments Received for the Record, Commencing with the January Number.*

MISSISSIPPI.—Dr. F. L. Fielder, $2 50; Dr. J. M. Taylor, 2 50; Dr. L. C. Harvey, 2 50; Dr. W. B. King, 2 50; Dr. L. S. Brownlee, 2 50; Dr. W. J. Harris, 2 50; Dr. J. H. Knott, 2 50; Dr. James S. Knott, 2 50; Dr. J. B. Bailey, 2 50.

GEORGIA.—Dr. J. W. Anthony, $2 50; Dr. S. B. Cousins, 2 50; Dr. W. H. Wilson, 2 00; Drs. Liddell and Richardson, 2 50; Dr. R. O. Lovitt, 2 50.

ALABAMA.—Dr. B. J. Foster, $2 50; Dr. J. R. Johnson, 2 50; Dr. J. C. Knox, 2 50; Dr. W. F. Jorden, 2 50; Dr. A. W. Agnew, 2 50.

TENNESSEE.—Dr. George Lacey, $2 50; Dr. M. J. Lewis, 2 00; Dr. G. H. Burnell, 2 00.

LOUISIANA.—Dr. Lockwood Alison, $2 50; Dr. S. L. Singletary, fifty cents, balance.

SOUTH CAROLINA.—Dr. G. W. Duval, $2 50.

ILLINOIS.—Dr. W. M. Garrard, $2 50.

OREGON.—Dr. J. L. Martin, $2 00.

TEXAS.—Dr. W. S. Posey, $1 00.

### The Southern Medical Record.

EDITED BY

T. S. POWELL, M.D.,

AND

W. T. GOLDSMITH, M.D.

### ASSOCIATE EDITORS:

GEORGIA.

Robert Battey, M.D.
R. C. [illegible]ord, M.D.
W. L. Kirkpatrick, M.D.
James A. Long, M.D.
W. L. M. Harris, M.D.
H. L Battle, M.D.
W. C. Musgrove, M D
L B. Bouchelle, M.D.
John C. Drake, M.D.
E. F. Knott, M.D.
Thomas Burdell, M.D.
E. H. W. Hunter, M.D.
V. S. Hitt, M.D.
J. E. Pope, M.D.
J. A. Hunnicutt, M.D.
A. H. Brantley, M.D.
J. J. Knott, M.D.
J. C. Wisdom, M.D.
W. W. Leake, M.D.

ALABAMA.

J. C. Knox, M.D.
Geo. F. Taylor, M.D.
J. B. Barnett, M.D.
S. M. Hogan, M.D.
D. S. Hopping, M.D.
J. T. Searcy, M.D.
S. F. Hill, M.D.

MISSISSIPPI.

C. B. Gallaway, M.D.
Edward Lea, M.D.
S. V. D. Hill, M.D.
W. B. Harvey, M.D.
J. W. M. Shattuck, M.D.
E. T. Henry, M.D.
T. P. Lockwood, M.D.
Robert Kells, M.D.

VIRGINIA.

Charles S. Lewis, M.D.
John H. Claiborne, M.D.
F. Horner, Jr., M.D.
R. J. Preston, M.D.
A. S. Payne, M.D.
J. E. Lockridge, M.D.
J. S. Wharton, M.D.
Wm. O. Owen, M.D.
Sam'l P. Ripley, M.D.
Benj. Blackford, M.D.
E. A. Drewry, M.D.
Rob B. Tunstall, M.D.
A. J. Terrell, M.D.
W. F. Barr, M.D.
L. B. Anderson, M.D.
S. L. Jepson, M.D., W Va.
J. M. Lazzell, M.D.

SOUTH CAROLINA.

E. T. McSwain, M.D.
G. M. Rivers, M.D.

TENNESSEE.

J. D. Tucker, M.D.
Swan M. Burnett, M.D.

TEXAS.

S. M. Welch, M.D.
T. J. Heard, M.D.
W. A. Heard, M.D.
A. R. Kilpatrick, M.D.

ARKANSAS.

A. D. Hodge, M.D.
J. K. Hodge, M.D.

NORTH CAROLINA.

J. R. Hughes, M.D.
S. S. Satchwell, M.D.
A. B. Pierce, M.D.
H. Kelly, M.D.
Geo. A. Foote, M.D.
E. A. Anderson, M.D.
H. T. Bahnson, M.D.
Willis Alston, M.D.
Thos. F. Wood, M.D.
N. J. Pittman, M.D.

### CORRESPONDING EDITORS:

J. M. Toner, M.D., Washington City, D. C.
John H. Weir, M.D., Illinois.
Thomas J Gallaher, M.D, Pennsylvania.
Ely Van DeWarker, M.D., New York.
Robert Robson, M D., Indiana.
C. C. F. Gay, M.D, New York,
*Surgeon of Buffalo General Hospital.*
W. T. Taylor, M.D, Kentucky.
J. Orne Green, Boston, Massachusets.
B. W. Stone, M.D., Kentucky,
*Assistant Physician Western Lunatic Asylum.*
James Tyson, M.D., Philadelphia, Pa.
W. W. Leen, M.D., Philadelphia.

M. H. Henry, M.D., New York,
*Surgeon to New York Dispensary, Department of Venerial and Skin Diseases.*
Wm. R. Whitehead, M.D., Colorado Territory.
A. Patton, M.D., Indiana.
Benjamin Howard, M.D., New York.
Chas Kearns, M.D., Kentucky.
S. C. Busey, M.D., Washington, D. C.
A. W. Hobson, M.D., Arkansas.
A. W. Calhoun, M.D., now of Berlin, Prussia.
T. Curtis Smith, Ohio.
Geo. Strawbridge, M.D., Philadelphia.

☞ *All Business Letters should be addressed to POWELL & GOLDSMITH, Post Office Box* 527, *Atlanta, Georgia.*

